# Controllable Microfluidic Production of Drug-Loaded PLGA Nanoparticles Using Partially Water-Miscible Mixed Solvent Microdroplets as a Precursor

**DOI:** 10.1038/s41598-017-05184-5

**Published:** 2017-07-06

**Authors:** Jiang Xu, Shusheng Zhang, Anais Machado, Sébastien Lecommandoux, Olivier Sandre, Frank Gu, Annie Colin

**Affiliations:** 1 Centre de Recherche Paul Pascal, CNRS, Univ. Bordeaux, 115 Avenue Schweitzer, 33600 Pessac, France; 2 Department of Chemical Engineering, Waterloo Institute for Nanotechnology, University of Waterloo, 200 University Avenue West, Waterloo, ON N2L 3G1 Canada; 3 CNRS, Solvay, LOF (UMR 5258), Univ. Bordeaux, F-33600 Pessac, France; 4 CNRS, Univ. Bordeaux, Bordeaux-INP, Laboratoire de Chimie des Polymères Organiques (UMR5629), 16 Avenue Pey Berland, 33607 Pessac, France; 5 ESPCI Paris, PSL Research University, Sciences et Ingénierie de la matière Molle, CNRS(UMR 7615), 10, Rue Vauquelin, 75231 Paris Cedex 05 France

## Abstract

We present a versatile continuous microfluidic flow-focusing method for the production of Doxorubicin (DOX) or Tamoxifen (TAM)-loaded poly(D,L-lactic-co-glycolic acid) (PLGA) nanoparticles (NPs). We use a partially water-miscible solvent mixture (dimethyl sulfoxide DMSO+ dichloromethane DCM) as precursor drug/polymer solution for NPs nucleation. We extrude this partially water-miscible solution into an aqueous medium and synthesized uniform PLGA NPs with higher drug loading ability and longer sustained-release ability than conventional microfluidic or batch preparation methods. The size of NPs could be precisely tuned by changing the flow rate ratios, polymer concentration, and volume ratio of DCM to DMSO (VDCM/VDMSO) in the precursor emulsion. We investigated the mechanism of the formation of NPs and the effect of VDCM/VDMSO on drug release kinetics. Our work suggests that this original, rapid, facile, efficient and low-cost method is a promising technology for high throughput NP fabrication. For the two tested drugs, one hydrophilic (Doxorubicin) the other one hydrophobic (Tamoxifen), encapsulation efficiency (EE) as high as 88% and mass loading content (LC) higher than 25% were achieved. This new process could be extended as an efficient and large scale NP production method to benefit to fields like controlled drug release and nanomedicine.

## Introduction

Poly(D,L-lactide-*co*-glycolide) (PLGA) nanoparticles (NPs) and microparticles exhibit great potential for nanobiomedicine^1^ as they are made of one the most commonly used biopolymers approved by the FDA due to their biosafety, biocompatibility, and biodegradability^2^. Currently, two methods are most frequently used for producing drug-loaded NPs: single (SE) or double emulsion (DE) methods and nanoprecipitation.

In the SE method^3^, the drug is dissolved in a mixture of an organic solvent and polymers and then emulsified in water. The use of a polar partially soluble organic solvent in water can promote the emulsification process^4^. In the DE method, the drug is dissolved in water and emulsified into an oily phase that contains the polymer. Then, the inverse emulsion is emulsified into an aqueous solution containing another polymer. The organic solvent is removed under vacuum. Both methods^3, 4^ enable the preparation of nanometric nanoparticles (typical size of 300 nm, size polydispersity index between 0.2 and 0.5) with a high encapsulation efficiency (EE) (usually higher than 67%) but a low drug loading content (LC) (usually smaller than 5%). The drug loading content is defined as the ratio of the mass of the drug divided by the mass of the polymer carrier. The encapsulation efficiency is the mass of the drug encapsulated in the nanoparticles divided by the mass of the drug initially present in the polymer-solvent mixture. For nanoparticles prepared using both kinds of emulsion methods, the drug release follows two-phase kinetics. The first phase (spanning over the first five hours) is characterized by drug diffusion through the superficial layers of the polymeric particles. This phenomenon is more important for particles prepared using the DE method. More than 7% of the drug content is released during this period for the double emulsion method compared to 1% for the simple emulsion method. This suggests that the superficial layers of nanoparticles prepared using the DE method are enriched with drug compared to the ones made using the SE method.

The term nanoprecipitation^5^, also known as the “solvent-displacement” method, refers to a quite simple processing method for the fabrication of polymeric nanoparticles. It involves the precipitation of a dissolved material into nanoscale particles after exposure to a nonsolvent of the polymer that is miscible with the solvent. In contrast, emulsion methods use immiscible or partially miscible solvents. Nanoprecipitation enables the fabrication of submicronic particles (typically approximately 150 nm) with a polydispersity index ranging between 0.1 and 0.5^5^. It is also the method of choice for producing rigid (glassy) or semi-crystalline polymers. However, the encapsulation efficiency and loading content are usually less than 20%^6, 7^. The amount of drug present in the superficial layers is also often more than 80%, leading to the phenomenon of burst release^6^.

Microfluidic technology has been developed for synthesizing series of either organic or inorganic NPs^8, 9^ in the past decade because of its advantages compared with bulk methods, including homogenous reaction environments from a single batch^10^, enhanced reproducibility^11^, non-excessive consumption of expensive agents^12^, and high, steady and fast throughput motored by mechanical valves and pumps^13^. Two microfluidic methods inspired from the bulk methods are most frequently used for producing drug-loaded NPs: (1) biphasic, i.e., droplet-based flow focusing and (2) rapid mixing of a solution with a selective solvent or a nonsolvent leading to nanoprecipitation of a compound.

The particles prepared from droplets formed in microfluidic devices are large; their size range typically between 1 and 100 µm. They are also monodisperse, and their size polydispersity index is less than 0.05^11–17^. The encapsulation efficiency can be higher than 70%, and the drug loading content can be higher than 20%^15^. They^15^ released drugs more slowly than conventional polydisperse particles with a similar average size. The initial burst release of drugs observed with monodisperse, microfluidic particles is significantly smaller than that observed with corresponding conventional, polydisperse particles prepared by bulk emulsion techniques. However, the main drawback of these micron-scale particles is that they are oversized for intravenous injection.

In contrast, continuous flow microfluidics has also been used in the literature for rapid mixing and nanoprecipitation by mixing a water-miscible organic fluid with an aqueous solution. This can provide small NPs (10~100 nm)^18, 19^ with a size polydispersity index ranging between 0.1 and 0.4^18^. However, relatively low encapsulation efficiency (usually less than 50%)^10^ and mass loading (usually less than 10%) are generally achieved^10^. Efforts have been made to accelerate the state of the art. Valencia *et al*.^18^ accelerated micromixing in a twisted channel and synthesized hybrid PLGA-lipid-PEG NPs *via* a single-step, achieving a much smaller particle size range less than 100 nm; however, a low drug loading capability was achieved (less than 5 w/w %). Hung *et al*.^19^ used solvent evaporation inside a microfluidic device for PLGA-DMSO droplet fusion with a water drop instead of the direct convection of discrete and continuous fluids. They succeed in preparing monodisperse submicronic nanoparticles ranging between 70 and 400 nm with a polydispersity index of size less than 0.3. However, they did not report drug encapsulation data.

One great challenge and motivation is thus to design a system that would decrease the size of NPs without sacrificing their drug loading ability. Here, we propose using a partially water-miscible mixture of dimethyl sulfoxide (DMSO) and dichloromethane (DCM), instead of pure DMSO or pure DCM, as a dispersed phase in a continuous flow microfluidic platform. To the best of our knowledge, this strategy combining microfluidics and nanoprecipitation in water from a solution of polymer and drugs in a solvent mixture is original. According to solvent handbooks, DCM is miscible in any proportion in DMSO^20^. The solubility of DCM in water at 20 °C is equal to 1.7% by weight, and the mass density of DCM is equal to 1.33 g/cm^3^. In a coaxial glass capillary-based microfluidic channel, thin stable jets of this partially water-miscible precursor solution transform into microdroplets and then into NPs under the action of the faster diffusion of DMSO in water compared to DCM. Strikingly, this method leads to a high mass loading capability (LC) and high encapsulation efficiency (EE) for both the hydrophobic drug (tamoxifen) and hydrophilic drug (doxorubicin).

With tamoxifen, an EE value as high of 88% was measured. Compared to previous bulk mixing methods using similar organic phases^3, 4^, doxorubicin-poly (D,L-lactide-*co*-glycolide), acid terminated) DOX-PLGA NPs synthesized using this microfluidic method are smaller (90~160 nm) and size-tunable and exhibit a comparable EE (up to 66%) and higher LC (up to 26.3%) by adjusting the flowrates, polymer concentration and *V*_*DCM*_/*V*_*DMSO*_. The release properties are not affected by this procedure and remain similar to the ones obtained using nanoprecipitation from pure DMSO but with a smaller initial burst release^3, 4^. These characteristics imply that our approach provides a novel and valuable method for producing NPs with a tunable size range under precise control at the nanoscale level and with significant drug loading capability.

## Results

### Investigation of the flow behaviours

**Figure 1:**
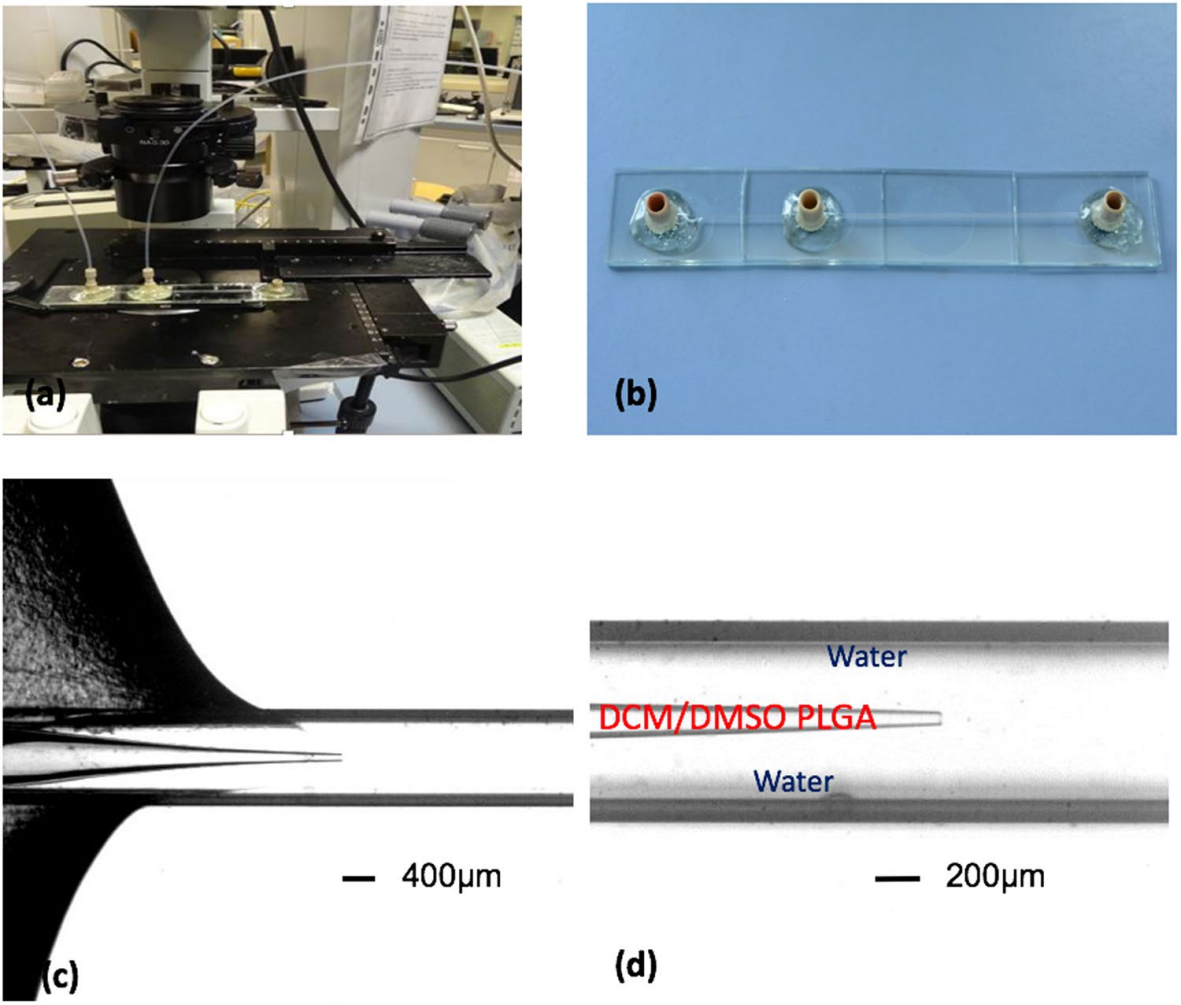
Coaxial microfluidic device fabricated with glass capillaries. (**a**) A microfluidic platform under operation; (**b**) A microfluidic chip constructed of glass capillaries and NanoPort assemblies (Upchurch Scientific); (**c**) The device under optical microscope; (**d**) The nozzle of the inside round capillary under optical microscope. The nanoparticles are formed away from the nozzle in the large capillary tube.

We used a classical coaxial flow microfluidic device (Fig. 1) constructed by following the protocol developed by the Weitz lab for obtaining self-centring capillaries^19^. A round glass capillary (0.50 mm ID × 0.70 mm OD, CM Scientific) was pulled by a micropipette puller and broken to form a ~50 µm ID tip (P-97, Sutter Instrument Company). (2) This tipped capillary was inserted into a square glass capillary (0.70 mm ID, CM Scientific) to obtain a coaxial geometry. The resulting coupled capillaries combined with the NanoPort assemblies (Upchurch Scientific) were stuck onto a glass platform.

**Figure 2:**
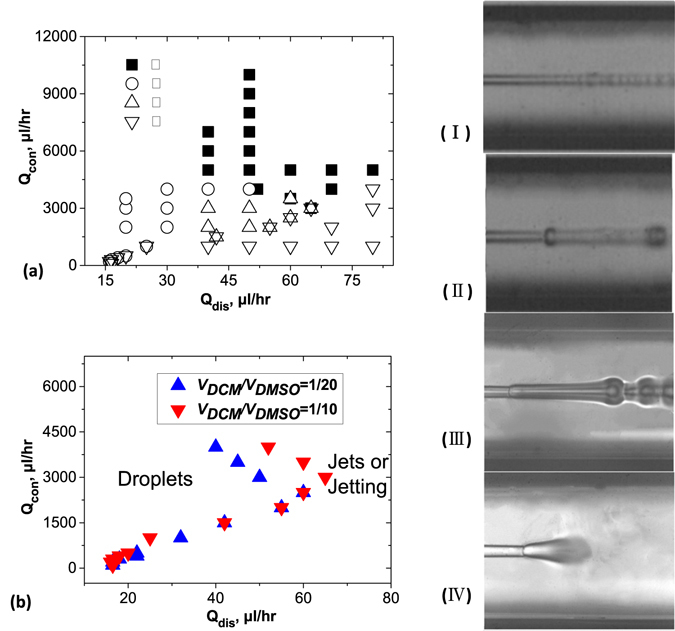
(**a**) Map of flow behavior in the (Qcon, Qdis) plane for fixed *V*_*DCM*_/*V*_*DMSO*_ = 1/10. Optical microscopy images (height: 870 µm) show different forms of the jet. Thin jets broke into tiny droplets at a well-defined location (I, solid squares). Other regimes led to droplets with periodic modulations: droplets with a diameter smaller than twice the nozzle size (II, open circles), and droplets with a diameter larger two times the nozzle size (III, open triangles). Finally, wide straight jets were stable at nozzle, and then diffused within water through the channel (IV, open inverted triangles). (**b**) Boundaries between droplets and jets shifted as the *V*_*DCM*_/*V*_*DMSO*_ was changed. Concentration of PLGA (Mw = 4000~15000 g/mol) = 5 mg/mL, in two DCM/DMSO mixtures (1/10 and 1/20 *v/v*). The boundaries of the flow diagram depend slightly upon the history of the experiment and upon the experiments. This explains why in some situations, we have reported two symbols for a given experimental condition.

Two syringe pumps (Harvard Apparatus PHD 4000) were used to drive the two fluid phases to flow coaxially in the same direction. The organic dispersed phase (5 mg/mL of PLGA (Mw = 4000~15000 g/mol) in a mixture of DCM/DMSO) flowed inside the round capillary, and the continuous phase (aqueous solution) flowed between the round and square capillaries. This led to coaxial injection at the tapered orifice. We observed flow patterns that varied significantly with the flowrates. Figure 2 displays different flow patterns for the partially water-miscible precursor solution with operational (Qdis, Qcon) flowrates close to the nozzle (i.e., at a distance shorter than the diameter of the external capillary tube). Qdis corresponds to the flow rate of the dispersed phase, and Qcon corresponds to the flow rate of the continuous phase. The patterns observed close to the nozzle were reminiscent of the ones obtained using immiscible fluids^21^. To be more precise, we noted no modifications in the flow diagram. A droplet regime was basically found for very low (Qdis, Qcon) flow rates, with droplets emitted periodically with sizes comparable to the nozzle radius (Fig. 2a-II, open circles) or larger droplets resulting from the instability of an emerging oscillating jet (Fig. 2a-III, open triangles)^20^. Large and short jets were found close to the nozzle (Fig. 2a-IV, inverted triangles). In this case, no visible macroscopic droplets were formed. For large external aqueous flow rates, we observed what we call jetting: thin and straight jets were produced (Fig. 2a-I, solid squares). The similarities between the partially miscible and not-miscible fluids are not surprising. Even though the fluids were partially miscible, they still exhibited weak interfacial tension. Jets of liquids that display surface tension are linearly unstable due to the Rayleigh-Plateau instability.

The nature of the instability at the linear level (absolute or convective) controls whether drops or jetting is obtained. In the convective case, growing disturbances are simultaneously transported downstream, and a continuous jet can persist in the system over some distance (which does not preclude the formation of droplets downstream). In contrast, in the absolute instability regime, the jet regime is never stable, as any perturbation generates oscillations that grow and travel backwards to invade the whole capillary. This behaviour corresponds to the regimes of the droplets and plugs. In our study, this process might be altered by the release of DMSO in water due to molecular diffusion. This is in fact not the case close to the nozzle. Because the Péclet number (Pe) (i.e., the ratio of the diffusion time to the convection time) is very large, diffusion plays a minimal role in the process, and the liquids behave as if they were immiscible. The Péclet number is given by equation 1, where Q is the flowrate, D is the molecular diffusion coefficient, and R is the size of the capillary tube. Typical values are Q = 2000 µL/hr, D = 210^−9^ m^2^/s^22^ and R = 700 µm, leading to Pe equal 1600, which is much larger than 1.

1 $${\rm{Pe}}={\rm{Q}}/{\rm{DR}}$$

The boundaries between the various zones were controlled be using a larger amount of DCM in the DMSO/DCM mixture (see Fig. 2b). At a given Qcon >2500 µL/hr, the precursor solution with the higher *V*_*DCM*_ */V*_*DMSO*_ ratio exhibited a droplet regime, while the one with a lower *V*_*DCM*_ */V*_*DMSO*_ ratio exhibited a jet regime at the same value of Qdis because the latter contained more of the insoluble component. This caused a higher interfacial tension and thus a larger drop regime domain in the parameter plane (Qdis, Qcon).

At first sight, our strategy thus seemed inefficient for producing small nanoparticles since the flow diagram was unchanged. However, the originality of our work and the possibility to reach our goal relied on the particle evolution after long durations. The jetting and droplets shrank as a function of time, i.e., as a function of their displacement in the capillary tube. When using the polymer solution instead of the pure solvent mixture, the jetting and droplet shrinkage induced the formation of submicron particles and, eventually, nanoparticles (NPs).

Using dynamic light scattering (DLS) (see Table 1), we noted that both the Z-average hydrodynamic size and polydispersity index (PDI) of the PLGA NPs (as deduced from a 2^nd^ order cumulant fit of the correlograms) decreased gradually with an increasing Qcon. PLGA NPs from the jetting region (region I) exhibited the advantages of having a smaller size with a narrower distribution and a higher throughput capability compared with other regions. Consequently, we focused on jetting region I for the rest of the study.

**Table 1:** Hydrodynamic diameter and PDI measured by DLS characterizing the broadness of the size distribution of PLGA NPs collected from the reservoir.

Region	I: Qdis = 50 µl/hr Qcon = 6000 µl/hr	II: Qdis = 50 µl/hr Qcon = 4000 µl/hr	III:Qdis = 50 µl/hr Qcon = 3000 µl/hr	IV:Qdis = 50 µl/hr Qcon = 1000 µl/hr
Hydrodynamic Diameter (nm)	103	106	128	321
Polydispersity Index	0.03	0.08	0.10	0.27
PDI width (nm)	15	31	41	167

Concentration of PLGA (Mw = 4000~15000 g/mol) = 5 mg/mL in DCM/DMSO mixture, *V*_*DCM*_/*V*_*DMSO*_ = 1/10. The PDI width is the standard deviation of size distribution computed from the square root of the PDI times the average hydrodynamic diameter.

### NP generation in the jetting zone: jet-to-droplets-to-nanoparticles

**Figure 3:**
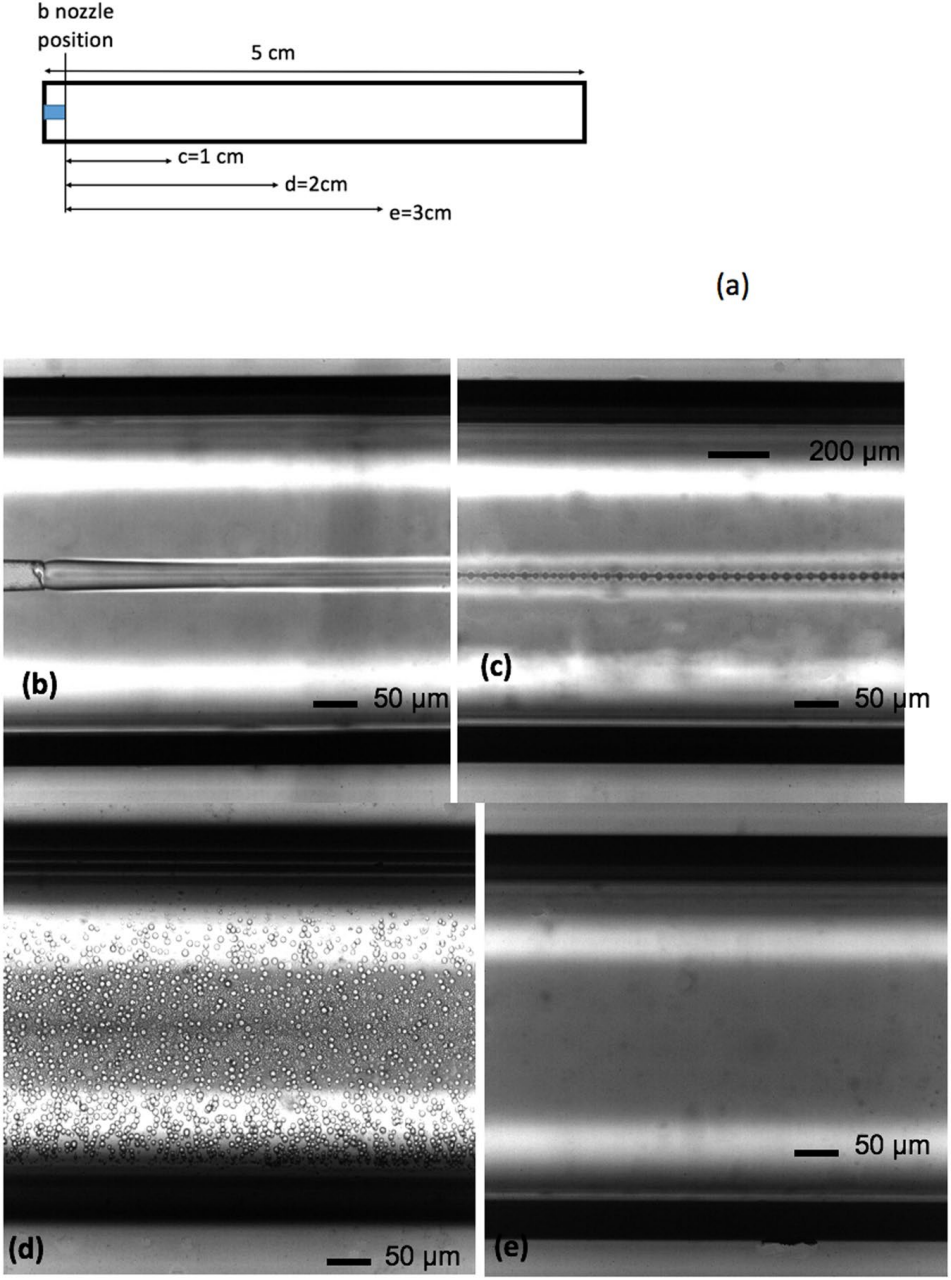
The evolution processes of different precursor fluids. (**a**) Scheme of the device with locations of the position for the images (b–e); (**b**–**e**) Flow-focusing of partially water-miscible precursor (*V*_*DCM*_/*V*_*DMSO*_ = 1/10) and water, Qcon = 10000 µL/hr, Qdis = 100 µL/hr. Photos were taken at positions close to nozzle (**b**), 10 mm after nozzle (**c**), 20 mm after nozzle (**d**), and 30 mm after nozzle (**e**), along the flow direction, successively. After 30 mm, the size of the droplets is submicronic since they are not observable using an optical microscope.

During traditional flow-focusing using water and water-miscible fluids, such as DMSO (Fig. 3a), nanoparticles–but not droplets–form after rapid mixing and nanoprecipitation since there is no interfacial tension between these two fluids, as has been well documented. However, in our experiments, when a water-immiscible component, such as DCM, was added to DMSO, making the mixed fluid partially water-miscible, non-vanishing interfacial tension was created; however, it was too low to be measured using the pendant drop technique. Then, a unique phenomenon was observed: a jet of fluid diffused into the water (Fig. 3b) and was gradually perturbed into a steady stream of homogenous droplets (Fig. 3c), which spread throughout the whole microfluidic channel from the restricted central jetting zone. Without sticking, and still flowing (Fig. 3d), these microdroplets finally shrunk into invisible nanoparticles under an optical microscope (Fig. 3e), yet they were detectable using DLS techniques. In the intermediate region of the capillary, the sizes of the initial droplets were between 1 and 3 microns depending on the flowrates.

Close to the outlet of the micro device, the particles were submicronic and droplets were not observed. The residence time through the capillary (tr) was shorter than 52 s, as estimated using equation 2, where a is the size of the squared capillary tube, l is the length of the channel, and Q is the total flow rate.

2 $${\rm{tr}}={{\rm{a}}}^{2}{\rm{l}}/{\rm{Q}}$$

DLS experiments were performed five min after the output of the product indicated the presence of nanoparticles. We estimated that the time required to produce the nanoparticles was between 52 s and 6 min.

### Effects of the polymer concentration, flowrate ratio, and *V*_*DCM*_/*V*_*DMSO*_ ratio on the size of the PLGA NPs in the jetting zone of the fluid

We further investigated the effects of different control parameters, specifically, the polymer concentration, flowrate ratio, and *V*_*DCM*_ */V*_*DMSO*_ ratio of the dispersed phase on the size of the resulting PLGA NPs.

**Figure 4:**
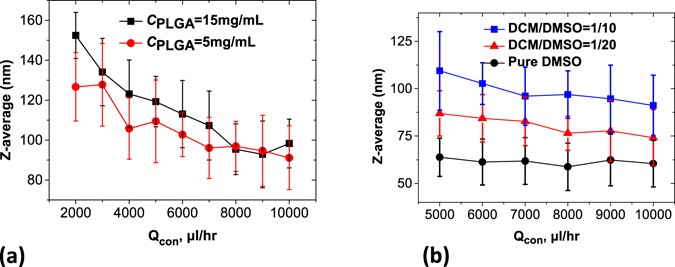
Hydrodynamic size of PLGA NPs synthesized with precursors of different flow ratios and different *V*_*DCM*_ */V*_*DMSO*_. Q_dis_ = 50 µL/h, Concentration of PLGA (Mw = 4000~15000 g/mol) = 5 mg/mL. Error bars are computed from the PDI width obtained by the 2^nd^ order Cumulant fit of the raw DLS curves.

From Fig. 4a, overall, a higher PLGA concentration formed larger nanoparticles. Noticeably, with an increasing flowrate, the NP volume difference between the high and low concentrations became smaller. With an increasing polymer concentration, the NP volume increased by a factor of ~1.5 to ~1.0 depending on the flowrate, which is much less than the concentration difference by a factor of 3 between these two curves. This trend indicates that our microfluidic technology exhibits good controllability over the particle sizes, especially at high flow rates, the mechanism of which is going to be discussed in the next section.

Furthermore, unlike the pure DMSO case where the NP size is independent of the Qdis/Qcon ratio, in Fig. 4b, the presence of DCM in the jetting zone causes a trend for larger (respectively lower) sizes associated with the lower (respectively higher) water flowrate. When the insoluble component in the precursor solvent increases (corresponding to *V*_*DCM*_/*V*_*DMSO*_ varying from 0 to 1/20 and 1/10), the size of PLGA NPs exhibited a positive correlation with *V*_*DCM*_ at fixed flowrate ratios, similar to the polymer concentration effect on the resulting particle size. Decreasing the Qdis/Qcon ratio (from 50/5000 µL/hr to 50/10000 µL/hr) caused the NP diameter to decrease from 64 ± 10 nm to 60 ± 12 nm (*V*_*DCM*_ = 0), 87 ± 12 nm to 74 ± 15 nm (*V*_*DCM*_ = 1/20), and 109 ± 21 nm to 91 ± 16 nm (*V*_*DCM*_ = 1/10), respectively (Fig. 4b). This occurred with an increase in the sizes of the originated droplets when more DCM was added.

### Mechanism of nanoparticle formation

**Figure 5:**
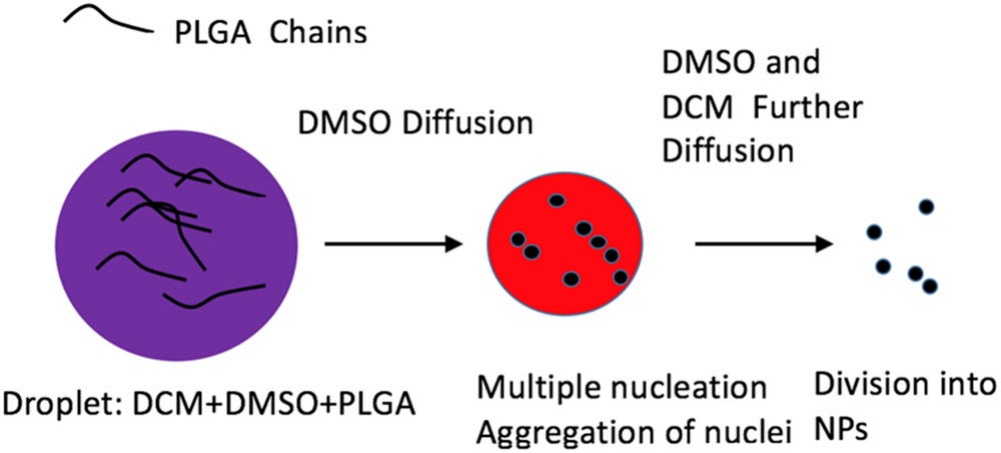
Mechanism of PLGA NPs formation from droplets. Purple color in the left circle represents the mixture of DCM + DMSO; Red color in the middle circle represents the mixture of DCM + DMSO, yet more DCM content; Solid black dots on the right side stand for PLGA NPs presumably still swollen by DCM, until its complete evaporation.

The previous observations allowed us to capture the salient features of the nanoparticle formation. This mechanism consists of three stages, having features of both conventional nanoprecipiation^8, 23, 24^ and emulsion-based flow focusing^25^ (see Fig. 5): (1) the more soluble component (DMSO) diffuses in the water, (2) the surface tension of the precursor solution increases gradually since the lower solubility component (DCM) becomes enriched in the droplets and, as a result, a stream of tiny microdroplets is generated in place of the jet, diffusing away from the nozzle. This process is explained by the Plateau-Rayleigh instability, by which surface tension causes the fluid stream to undulate and eventually break into a train of droplets. The value of the interfacial tension driving this process is very difficult to obtain. In our experiments, the surface tension was not in equilibrium and exhibited dynamic surface tension during mixing. Measuring such a transient surface tension between the miscible fluids is out of the scope of this paper. Furthermore^26^, its value is higher than 10^−5^ mN/m. The pure DCM displayed an interfacial tension equal to 27.1 mN/m with water (the value was measured using a pendant drop), while it became 4.7 mN/m in the DMSO-DCM mixture with *V*_*DCM*_/*V*_*DMSO*_ = 4/10 (see Supporting Information Figure S8). We thus consider that the dynamic surface tension is between these limiting values. We also anticipate that the dynamic interfacial tension increases when the amount of DCM increases from 0 to 1/10 or 2/10. (3) During the third and last parts of the process, both the DCM and remaining DMSO diffused in the water. We recall that due to the non-zero solubility of DCM in water, when the water flowrate was higher than 7.8 times the solvent flowrate, the total amount of introduced DCM was soluble in water for DCM/DMSO = 1/10 (respectively 3.9 times that for DCM/DMSO = 1/20). This condition was fulfilled in region A. During the diffusion of the two solvents, the size of the droplets decreased, and the concentration of the polymer increased and finally reached the concentration corresponding to the solubility of the polymer in DCM. Precipitation thus occurred, and the NPs appeared as solid nuclei. At this stage, we wondered if a single droplet created a single nanoparticle or many of them. If we assume that one single originating droplet corresponds to a number (*N*) of formed nanoparticles in the final stage, we can calculate the mass of the PLGA contained in the NPs given by equation 3:

3 $${c}_{o}\frac{4}{3}\pi {R}^{3}=N{\rho }_{f}\frac{4}{3}\pi {r}^{3}\,$$

where *c*_*o*_ is the original polymer concentration, *c*_0_ = 5 mg/mL = 0.005 g/cm^3^; *ρ*_f_ stands for the final polymer mass density; *R* stands for the radius of the original droplet ≈ 1.5 µm; *r* stands for the radius of the final nanoparticle ≈50 nm; and *ρ*_*f*_ is the final density ≈1.25 g/cm^3^ from the handbook value for amorphous PLGA). Using this simple equation, one can calculate that a single droplet will eventually separate into a number of nanoparticles equal to *N* = *c*_*o*_ *R* ^3^/*ρ*_*f*_ *r* ^3^ ≈ 100. From these simple calculations, we can thus conclude that NP formation^27^ occurs through the classical nucleation-growth mechanism, also called the Lamer model. However, from the analysis of the NP size dependence as a function of the polymer concentration, we conclude that during the circulation time in the chip, the NP growth is negligible compared to their nucleation. Increasing the concentration of the polymer indeed changes neither the size of the original droplets nor the size of the resulting NPs. This indicates that when the concentration increased, the polymer chains were inclined to form more numerous nuclei, thereby generating more NPs rather than growing into larger NPs through a ripening process, which is consistent with the observation in Fig. 4a. Such separation of nucleation and growth is often reported as a clear advantage of NP syntheses in microfluidic chips^28^. This characteristic proves that the nuclei appear very rapidly and then aggregate through the DLCA (diffusion-limited cluster aggregation) mechanism^27^, producing smaller and monodisperse NPs, due to the rapid diffusion of both DCM and DMSO into water.

Let us stress that the monodispersity of the obtained NPs is a feature of the fast nucleation followed by the DLCA mechanism^25^. This mechanism captures the dependence of the NP sizes on the DCM content. Adding more DCM to DMSO in the solvent increases the dynamic surface tension between the fluid and water, thereby increasing the sizes of the originating droplets. The larger the originating droplet is, the longer the time it takes to shrink^26^. Under these slow diffusion conditions, the concentration of the polymer chains inside the droplets does not reach the supersaturation threshold and much fewer nuclei are formed. Then, polymers can easily adsorb on them, starting a growth mechanism concomitantly with further nucleation. Increasing the amount of DCM thus promotes growth and larger (but less monodisperse) nanoparticles.

### Drug encapsulation and release using microfluidics

**Figure 6:**
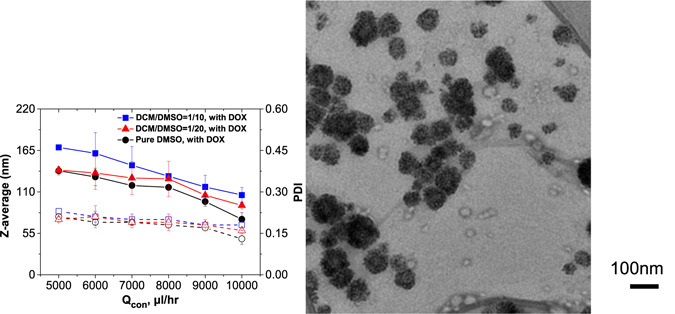
Size of DOX-encapsulated PLGA NPs synthesized with precursor solvents of different flowrate ratios and different compositions *V*_*DCM*_ */V*_*DMSO*_. The solid markers represent the particle hydrodynamic sizes and the open ones the polydispersity index (PDI) as deduced by 2^nd^ order Cumulant fit of the correlogram curves. Qdis = 50 µL/hr. Concentration of PLGA (Mw = 4000~15000 g/mol) = 5 mg/mL. Concentration of Doxorubicin = 1 mg/mL. (n = 3; mean ± S.D.). TEM image of DOX-PLGA NPs (the scale bar is 100 nm) to demonstrate spherical shape of the nanoparticles.

The nanoparticle formation mechanism was not affected by the introduction of drugs in the organic solution. Hydrophilic drugs, such as DOX, precipitate because DMSO ripening rapidly induces bad solvent conditions in the DCM-rich droplets. Although the DOX and polymer may precipitate separately, they form hybrid spherical NPs together. Indeed, the TEM images (see Fig. 6) evidence the presence of dark points (DOX) on a grey background (PLGA). The nuclei of the polymers and drugs were thus dissociated and aggregated following the DLCA process, as previously discussed.

As shown in Fig. 6, the sizes of the DOX-loaded NPs varied as a function of the flowrate ratios and solvent compositions (*V*_*DCM*_ */V*_*DMSO*_) in a consistent manner compared to the NPs without drugs (Fig. 4). Noticeably, the DOX-PLGA NPs still exhibited a low size dispersity, with a polydispersity index that gradually decreased from 0.2 to 0.15 as the flow ratio (Qdis/Qcon) decreased, which proves that rapid mixing within a flow-focusing mixing microfluidic chip offers a more homogenous environment than bulk nanoprecipitation for drug-loaded NP formulation. Drug encapsulation efficiency (EE) is defined as the fraction of the initial DOX that is encapsulated within the PLGA NPs compared to the initial feeding content, whereas the mass loading capability (LC) is defined as the mass fraction of the encapsulated drug compared to the polymer weight in the nanoparticles. In Table 2, both the EE and LC increased with increasing *V*_*DCM*_ */V*_*DMSO*_, regardless of the flowrate ratio. With the addition of DCM from 0 to 1/20 and to 1/10, the EE increased from 48.5% to 49.9% and 56.9%, respectively; meanwhile, the mass loading capability (LC) increased from 9.7% to 10.0% and to 11.4%, respectively, at a low flow ratio (Qdis/Qcon = 50/10000).

**Table 2:** Comparison of drug encapsulation efficiency (EE) and loading capability (LC) of PLGA NPs prepared at different flowrate ratios, compositions *V*_*DCM*_ */V*_*DMSO*_, and initial feeding drug concentrations.

Drug	Drug Feed (mg/mL)	Flow ratio (Qdis/Qcon, µL/hr)	*V*_*DCM*_ */V*_*DMSO*_	EE (%)	Mass Loading Capability LC (%)	Diameter+/−S.D. (nm)
DOX	1	50/10000	0/10	47 ± 2	9.5 ± 0.5	74 ± 9
DOX	1	50/10000	1/20	49 ± 11	10 ± 2	92 ± 4
DOX	1	50/10000	1/10	56 ± 15 (23 ± 0.5)*	11 ± 3 (4.5 ± 0.1)*	106 ± 10 (153 ± 4)*
DOX	1	50/5000	0/10	57 ± 4	11 ± 1	138 ± 5
DOX	1	50/5000	1/20	74 ± 8	15 ± 2	138 ± 3
DOX	1	50/5000	1/10	80 ± 2 (60 ± 3)*	16 ± 0.3 (12 ± 0.5)*	169 ± 2 (202 ± 8)*
DOX	2	50/10000	1/10	51 ± 3	21 ± 1	188 ± 5
DOX	2	50/5000	1/10	65 ± 4	26 ± 2	227 ± 3
TAM	1	50/10000	1/10	88 ± 6	18 ± 1	196 ± 4
TAM	1	50/5000	1/10	77 ± 5	15.5 ± 1	211 ± 5

Concentration of PLGA (Mw = 4000~15000 g/mol) = 5 mg/mL. Asterisk symbol represents data by bulk method with same parameters. (n = 3; mean ± S.D.).

Consistently, the EE increased from 58.1% to 74.3% and to 79.8%, respectively; meanwhile, the mass loading capability increased from 11.6% to 14.9% and to 16.0%, respectively, at a high flow ratio (Qdis/Qcon = 50/5000). These results indicate that this partially water-miscible precursor fluid could help to preserve drugs within the NPs with better efficiency than using pure DMSO and other classical water-miscible fluids in the literature^29^ by introducing DCM. This phenomenon was attributed to the interfacial tension between the partially water-miscible fluid and water, as discussed in Section 2.2, which helped to “trap” the drug inside the NPs during their formation. Higher *V*_*DCM*_ */V*_*DMSO*_ ratios led to higher EE values, which was attributed to the fact that precursor solutions with higher *V*_*DCM*_ */V*_*DMSO*_ ratios had a larger dynamic interfacial tension with water; hence, more drug molecules could be loaded. In addition, it is also a well-known effect that DCM can play the role of a plasticizer that increases the mobility of the PLGA chains, which are otherwise in a glassy or even semi-crystalline state. The beneficial effect from adding a good solvent and plasticizer for the polymer chains has been described for the nanoprecipitation of vesicles from copolymers with glassy polystyrene blocks^30, 31^. In addition, both the EE and LC of the PLGA NPs were improved using microfluidics compared to bulk mixing protocols, especially at low flow ratios (Qdis/Qcon). This feature is most likely due to the superior controllability of microfluidics for manipulating tiny volumes of fluid at rapid mixing rates^32^. Note that the EE decreased when a higher drug feed concentration was used, which was certainly due to a lack of polymer molecules available to encapsulate the drugs.

We further investigated the effect of the initial DOX concentration on the LC and EE. With a fixed *V*_*DCM*_ */V*_*DMSO*_ = 1/10 at low flow ratio (Qdis/Qcon = 50/10000), after doubling the drug feed concentration from 1 mg/mL to 2 mg/mL, the mass loading capability greatly increased from 11.4% to 21.1%, a level rarely reached using polymer drug carriers (excluding polymer conjugates), and the EE slightly decreased from 56.7% to 52.7%. Similarly, at a high flowrate ratio (Qdis/Qcon = 50/5000), the mass loading capability greatly increased from 16.0% to 26.3%, and the EE just slightly decreased from 79.8% to 65.9%. The mass loading ratio shown for these PLGA NPs is not only significantly higher than that in previous studies using conventional preparation protocols^33, 34^ but also comparable to droplet-based microfluidic methods using a pure water-immiscible solvent^35, 36^, and the relatively high EE ensures minimal waste of costly and dangerous cytotoxic drugs. When we switched the drug from doxorubicin to tamoxifen, the affinity between the drugs and solvents had a great effect on the size and encapsulation capability of the NPs. The PLGA NPs containing tamoxifen had larger sizes (196 nm with a low flow ratio and 211 nm with a high flow ratio) compared to those containing doxorubicin (106 nm with a low flow ratio and 169 nm with a high flow ratio). Tamoxifen is more hydrophobic than doxorubicin; hence, its solubility in DCM is rather high. It precipitates after crossing of the solubility line during DCM diffusion in water. We assume that this was correlated to the appearance of a lower number of nuclei compared to doxorubicin (at the same time or distance relative to the nozzle) and proceeded through a comparable DLCA mechanism, which finally led to larger NPs, as discussed in Section 2.2. In addition, the NPs exhibited a significantly improved EE (from 57% to 89%) and LC (from 11% to 18%) with tamoxifen compared to doxorubicin at a low flowrate ratio (Qdis/Qcon = 50/10000). The enhanced behaviour was attributed to tamoxifen having a higher partition coefficient in DCM than doxorubicin so that less tamoxifen could diffuse alone into the water; hence, tamoxifen remained within the NPs after the solvent exchange.

**Figure 7:**
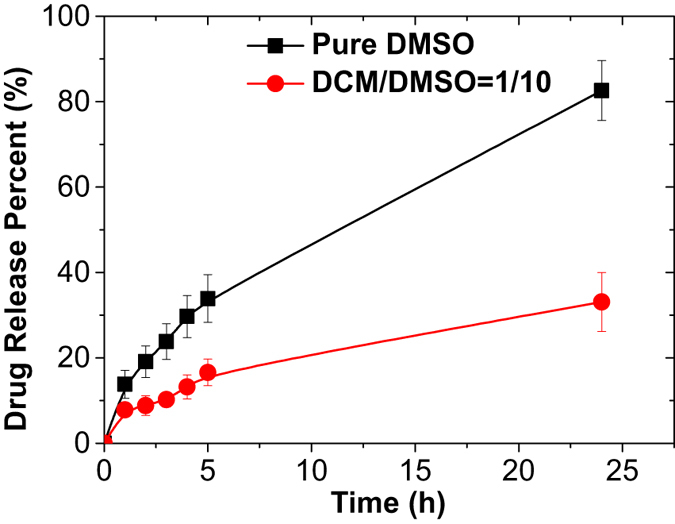
Comparison of the *in vitro* doxorubicin (DOX) cumulative release profiles using PLGA NPs obtained at different *V*_*DCM*_ */V*_*DMSO*_ ratios.

The role that *V*_*DCM*_ */V*_*DMSO*_ played during the drug release process of the previously formulated NPs was also studied. We found that the DOX-PLGA NPs containing DCM during their formation exhibited a slower and sustained release profile, compared to those obtained without DCM during preparation (Fig. 7). As previously suggested^37–39^, DCM is inclined to shrink towards the cores of the NPs during the evaporation process so that more drug is expected to be “dragged to” and embedded “deeper” in the hydrophobic cores of the NPs. This feature of the partially water-miscible precursor solvent implies that it could help to encapsulate more drugs inside the NPs to preserve their native and functional state and to facilitate a more sustained release profile. For example, after 24 h, only 33.1% of the doxorubicin was released from the DOX-PLGA NPs containing DCM during their formation, while 82.6% of the doxorubicin was released from the NPs without DCM present during their formation. Drug protection within the core of the NPs is very desirable for doxorubicin, which has been described to degrade within a few hours when solubilized in an aqueous medium^31^, and also for most drugs that might be sensitive and degraded during their biodistribution.

## Conclusion

Herein we have successfully developed a novel microfluidic continuous flow-focusing method for the preparation of drug-loaded and biodegradable DOX-PLGA and TAM-PLGA NPs using a partially water-miscible solvent mixture. We investigated the stability of the partially water-miscible solvent under the confinement of microfluidic channels. A novel transformation process for the precursor fluid in the jetting zone of the chip called the “Jet-to-Droplets-to-Nanoparticles” process was observed and described, for the first time. The PLGA NP size can be precisely tuned by controlling the flowrate ratio, polymer concentration, and solvent volume ratio (*V*_*DCM*_ */V*_*DMSO*_) of the dispersed phase.

Furthermore, the NPs synthesized using the partially water-miscible precursor solvent in the flow-focusing microfluidic chip exhibited much better drug loading capability and a longer sustained release stage than conventional methods using the classical nanoprecipitation method with a water-miscible organic solvent. To summarize, the success of our process relies on the formation of micrometric droplet emulsions due to the Rayleigh Plateau instability. The DCM, even though it displays an interfacial tension with water, is totally soluble in water using our utilized flowrate ratios. Fast ripening after solvent diffusion of the initial droplets due to their small sizes promoted a burst nucleation mechanism, followed by diffusion-limited cluster aggregation (DLCA), maintaining a low size-dispersity. This reduced the loss of drug into the water, promoted the monodispersity of the obtained NPs and ensured that the drug remained trapped in the core of the NPs.

In addition, our microfluidic method with a significantly high drug loading rate does not cause any clogging effects for the NPs or drug aggregation during the long-time flow-focusing process (several hours). All of these characteristics prove that this microfluidic flow-focusing method using a partially water-miscible solvent mixture is a clean, highly efficient, and robust platform for producing drug-encapsulating NPs, and it has great potential for further applications, such as in the cosmetics industry, controlled release, and 3D printing.

## Experimental Methods

### Materials

Dichloromethane (HiPerSolv CHROMANORM for HPLC, VWR) and dimethyl sulfoxide (ACS grade, Amresco) were mixed at volume ratios of 1/10 and 1/20 to prepare the precursor solutions. Poly (D,L-lactide-*co*-glycolide) (acid terminated) (75:25, Mw 4000~15000 g/mol, Sigma Aldrich) was dissolved in the dispersed phase at different concentrations (5 mg/mL, 15 mg/mL). Phosphate buffered saline (PBS, 150 mM, pH = 7.4 at 25 °C) was purchased from Sigma Aldrich. The doxorubicin (DOX) was supplied by Discovery Fine Chemicals (Wimborne, UK), and tamoxifen was supplied by Sigma Aldrich.

### Synthesis of PLGA nanoparticles using microfluidic flow focusing

The dispersed phase flowrate (Qdis) was varied between 10 to 100 µL/hr, and the aqueous flowrate (Qcon) was varied between 1000 and 10000 µL/hr. The solutions containing PLGA NPs were collected from the outlet and water-bath evaporated for 1 hour at 45 °C to remove the DCM solvent. The PLGA NPs were characterized using dynamic light scattering in backscattering mode at a 135° angle (Vasco™ DL135 particle size analyser, Cordouan Technologies, Pessac, France) after solvent evaporation. Each measurement was repeated 3 times (n = 3, mean ± S.D). The correlograms were analysed with the 2^nd^ order cumulant fitting method to yield the Z-average hydrodynamic diameter and polydispersity index (PDI), defined as the ratio of the 2^nd^ order coefficient to the square of the 1^st^ order coefficient in the cumulant series.

### Drug encapsulation using the PLGA nanoparticles

The doxorubicin (DOX) supplied by Discovery Fine Chemicals (Wimborne, UK) was commercially modified as doxorubicin hydrochloride. Therefore, triethylamine (TEA, ≥99.5%, Sigma-Aldrich) was used to incubate the DOX solution in DMSO overnight with a 1:2 molar ratio to deprotonate the molecule and render it more hydrophobic, as first described by Kataoka *et al*.^40, 41^. Then, the drug (DOX or tamoxifen, Sigma Aldrich) and PLGA were dissolved in the precursor solution at 1 mg/mL and 5 mg/mL, respectively.

Next, 1 mL of the suspension from the outlet of the microfluidic chip was collected from the capillary reservoir and then transferred to an Amicon™ ultrafiltration tube (MWCO = 3 kDa, Amicon™ Ultra-4) 30 min of centrifugation at 8000 rpm to separate the drug-loaded NPs from the buffer^42^. The molar mass of the DOX was 543 Da, and the molar mass of the TAM was 372 Da. Both drugs could thus easily pass through the ultrafiltration membranes. We used a single washing step in order to minimize the operation time before the drug release kinetics were measured. Note that the drug concentration measurements after one or two washing steps were found to be approximately the same, within an uncertainty margin (typically ± 5%).

We controlled flow rates of the two inlet flows precisely, in which the concentrations of the drug and polymer were known as we set them. From the values of the two flow rates, we calculated the mass of the polymer (mp) and drugs (md). To determine the encapsulation efficiency, we used ultrafiltration to remove any non-encapsulated drug from the solution, while the drug-loaded NPs were collected by a filter in the centrifuge tube. The washing procedure was straightforward. The solubility of DOX in water is 10 mg/mL, which is much higher than the solubility of tamoxifen (16.7 μg/mL). Neglecting the polymer and DMSO volumes, during the situation in which no drug was encapsulated, the drug concentration at the outlet of the device is equal to (Qdis/Qcon)*c, where c is the drug concentration in the dispersed phase. As Qdis/Qcon was less than 10^−2^, the concentration of the drug in water was less than 10 μg/mL. For both drugs loaded at c = 1 mg/mL, this concentration is below the solubility limit. No precipitates were formed, and the washing procedure was simple. Note that we did not perform multiple washing steps in order to avoid releasing the initially encapsulated drugs. The filtered NPs were resuspended in water for DLS measurements or dissolved in 1 mL of DMSO for encapsulated drug titration.

HPLC or UV/VIS and fluorescence absorbance measurements are appropriate methods for measuring drug contents in nanoparticle suspensions. As noticed by Schwendeman^43^, when the mass loading is large, i.e., in our situation, UV/VIS or fluorescence are sufficiently accurate and easy to use technics. Following this recommendation, and previous work^3^, we measured the encapsulation efficiency and drug loading content using UV/VIS absorbance measurements.

The percentage (x) of the doxorubicin in the polymer mixture was estimated by measuring the VIS absorbance of the solution at **λ** = 481 nm (SpectraMax M2 plate reader) and comparing the results to a calibration curve (see Supporting Information Figures S1, S2, S5 and S6 and Tables S1 and S3).

For tamoxifen, the filtered NPs were resuspended and dissolved in a 2 mL mixture of DMSO and methanol^44, 45^ (1:1 *v:v*), and the percentage (x) of TAM was determined by measuring the UV absorbance at **λ** = 285 nm (see Supporting Information Figures S3 and S4 and Table S2).

Each measurement was repeated 3 times (n = 3, mean ± S.D.). We deduced the loading content (x), which is equal to the ratio of md divided by mp, and the encapsulation efficiency (EE), which is the weight of drug in the NPs divided by the initial drug feeding weight. DOX and TAM being photosensitive drugs, we used black glass bottles to store the drug solution in order to avoid their degradation. To measure the encapsulation efficiency, we performed absorbance measurements during the first 5 minutes following the preparation of the sample. During the release experiments, in the lag time between the measurements, the samples were protected using aluminium foil.

### Drug Release by PLGA nanoparticles

The drug release is presented in Fig. 7. The DOX-PLGA NPs solution was collected at Qdis = 50 µL/hr and Qcon = 5000 µL/hr (C_DOX_ = 1 mg/mL, *V*_*DCM*_/*V*_*DMSO*_ = 1/10) for a total volume of 20 mL, which was concentrated to 1 mL using an ultra-centrifugal filter unit (4500 rpm ultracentrifugation, MWCO = 10 kDa, Amicon™ ultra15). The molar mass of DOX as 543 Da, and the molar mass of TAM was 372 Da. The drugs thus easily passed through the membranes. The concentrated NP suspension was then transferred into a dialysis membrane (MWCO = 25 kDa) and equilibrated against 40 mL of PBS solution (150 mM, pH = 7.4) in a release bottle with magnetic stirring at T = 37 °C. We chose a membrane with larger pores than the ones used during the washing step in order to measure the drug release kinetics from the particles while not being limited by the kinetics of the drug passage through the dialysis membrane. The cumulative released dose was determined using UV-VIS spectroscopy. A DOX-PLGA NP suspension made with the same protocols but using pure DMSO was used as the control group.

## Electronic supplementary material

Supplementary Info
