# Supplementary material for: Controllable Microfluidic Production of Drug-Loaded PLGA Nanoparticles Using Partially Water-Miscible Mixed Solvent Microdroplets as a Precursor
Source: Sci Rep. 2017 Jul 6;7:4794. doi: 10.1038/s41598-017-05184-5 (PMC5500499; doi:10.1038/s41598-017-05184-5)
Supplement: Supplementary file 1 — Supplementary Info [file 41598_2017_5184_MOESM1_ESM.pdf]

Supporting Information for

Controllable Microfluidic Production of Drug-loaded PLGA Nanoparticles Using Partially Water-miscible Mixed Solvent Microdroplets as Precursor

Jiang Xu[1,2,3], Shusheng Zhang[4], Anais Machado[4], Sébastien Lecommandoux[4], Olivier Sandre[4], Frank Gu[2], Annie Colin[1] *

[1]Centre de Recherche Paul Pascal, CNRS, Univ. Bordeaux, 115 Avenue Schweitzer, 33600 Pessac, France

[2]Department of Chemical Engineering, Waterloo Institute for Nanotechnology, University of Waterloo, 200 University Avenue West, Waterloo, ON, N2L 3G1, Canada

[3]CNRS, Solvay, LOF (UMR 5258), Univ. Bordeaux, F-33600 Pessac, France

[4]CNRS, Univ. Bordeaux, Bordeaux-INP, Laboratoire de Chimie des Polymeres Organiques (UMR5629), Pessac, France

[5] ESPCI Paris, PSL Research University, Sciences et Ingénierie de la matière Molle, CNRS(UMR 7615), 10, Rue Vauquelin, 75231 Paris Cedex 05, France

Each data point was measured by 3 times to calculate the average and standard deviation.

1. Determination of concentration of Doxorubicin in DMSO

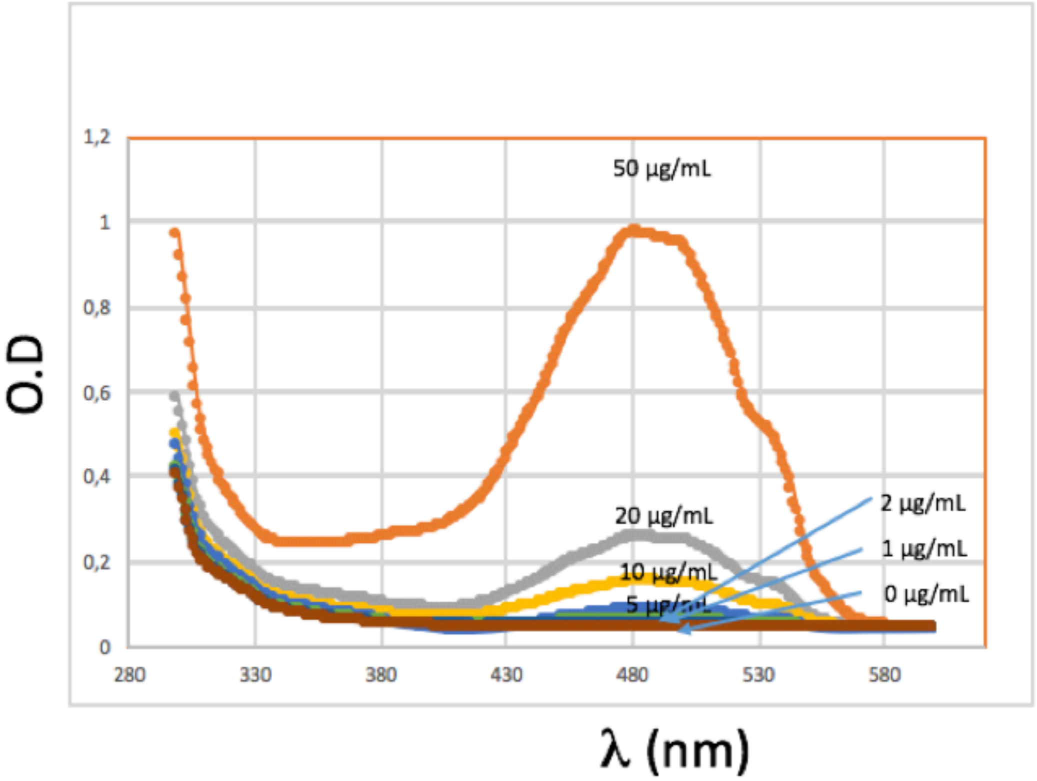

Figure S1: UV/Vis absorption of Doxorubicin in DMSO from 300 nm to 600 nm.

There is a strong VIS absorbance peak at 481nm and a shoulder near 530 nm. We thus selected the absorption values at 481nm as the characteristic VIS absorbance values. The VIS absorbance values @481nm for Doxorubicin samples of different concentrations in DMSO were measured to build a standard calibration curve. The thickness of the cuvette was kept at 1 mm. The blank solution corresponds to pure DMSO. We have checked that the presence of PLGA molecules in concentration lower than 200 µg/ml do not change the absorbance of pure DMSO.

Concentration of Doxorubicin,μg/mL	VIS absorbance at 481nm
50	0.970967
20	0.2564
10	0.1537
5	0.088634
2	0.0638
1	0.054634
0	0.046133

Table S1: VIS absorbance at 481 nm for various concentrations of Doxorubicin in DMSO.

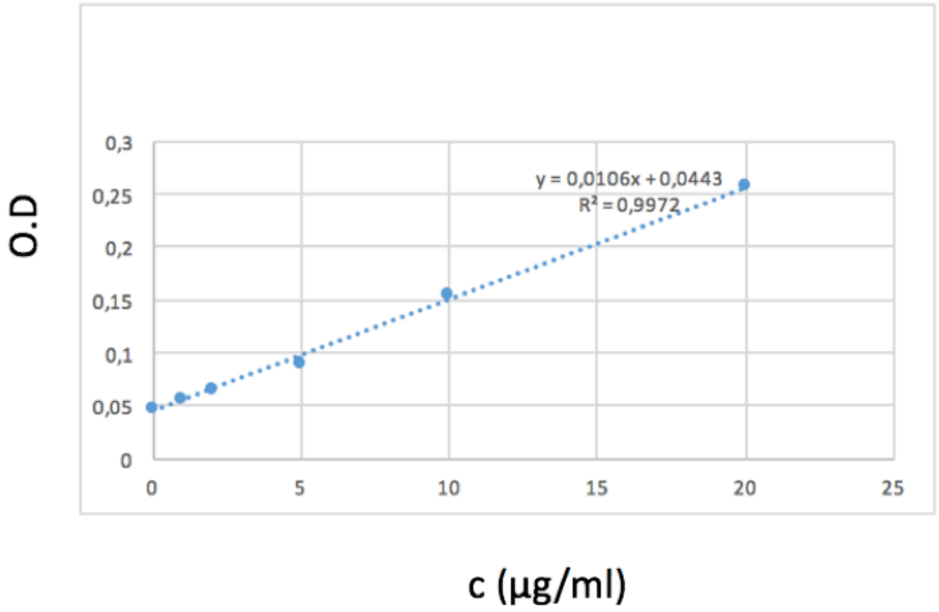

Figure S2: Standard calibration curve obtained from the absorption at 481 nm of Doxorubicin solutions in DMSO (cuvette length was 1 mm).

2. Determination of concentration of Tamoxifen in DMSO:Methanol mixture (1:1 v:v)

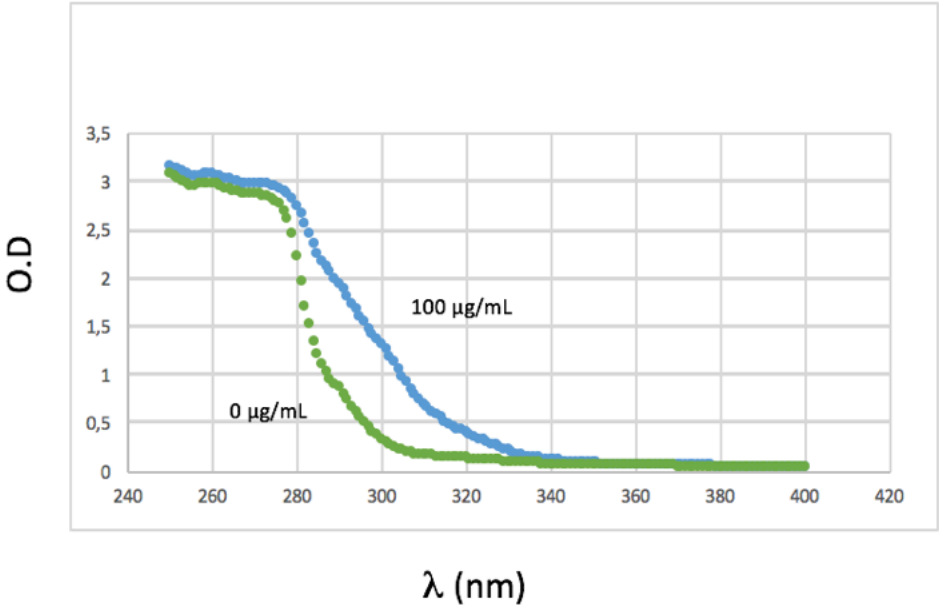

Figure S3: UV absorption of Tamoxifen in mixture of DMSO:Methanol (1:1) from 250 nm to 400 nm. The quartz cuvette transmits light above 280 nm only.

There is not significant UV absorbance peak ascribed to the drug. We selected the absorption values at 285 nm as the characteristic UV absorbance values, since it gives the largest variance following concentration variations.
We then measured the UV absorbance values @285 nm with Tamoxifen samples of different concentrations to build the standard calibration curve. The blank solution corresponds to DMSO:Methanol mixture (1:1 v:v). We have checked that the presence of PLGA molecules in concentration lower than 100 µg/ml do not change the absorbance of DMSO: :Methanol (1:1).

Concentration of Tamoxifen, µg/mL	UV absorbance at 285 nm
100	2.25946667
10	1.31916667
5	1.27555
2	1.2355
1	1.20896667
0	1.20346667

Table S2: UV absorbance at 285 nm for various concentrations of Tamoxifen in

mixture of DMSO:Methanol (1:1 v/v) mixture.

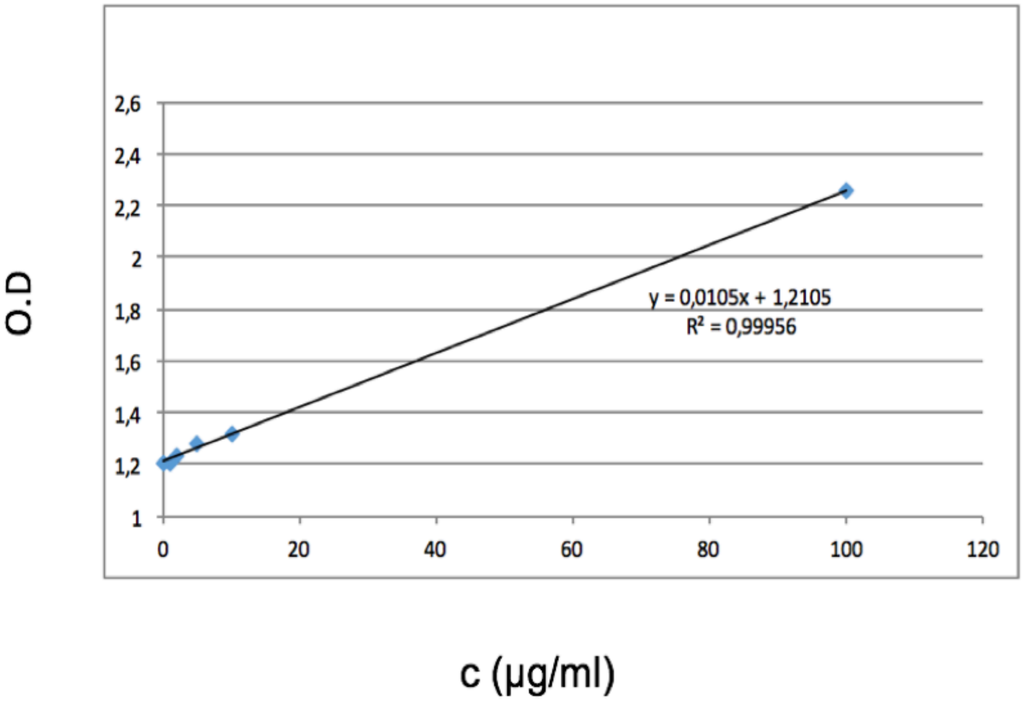

Figure S4: Standard calibration curve of UV absorption of Tamoxifen measured at 285 nm in DMSO:Methanol mixture (1:1 v:v)

3. Determination of concentration of Doxorubicin in PBS solution

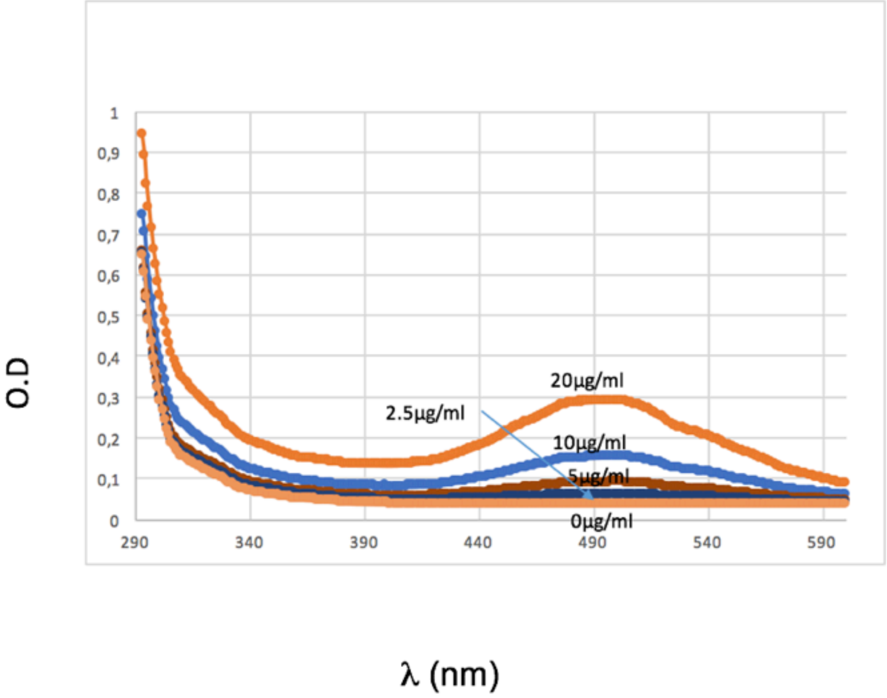

Figure S5: UV/Vis absorption of Doxorubicin in PBS from 290 nm to 590 nm.

There is a VIS absorbance peak at 499 nm. We then selected the absorption values at 499 nm as the characteristic VIS absorbance values.

We then measured the VIS absorbance values @499nm with Doxorubicin samples of different concentrations to build the standard calibration curve. The blank solution corresponds to pure PBS solutions. We have checked that the presence of PLGA molecules in concentration lower than 200 µg/ml do not change the absorbance of pure PBS solutions.

Concentration of Doxorubicin, μg/mL	VIS absorbance at 499 nm
20	0.2846
10	0.1517
5	0.091367
2.5	0.061333
0	0.03647

Table S3: VIS absorbance at 499 nm for various concentrations of Doxorubicin in PBS.

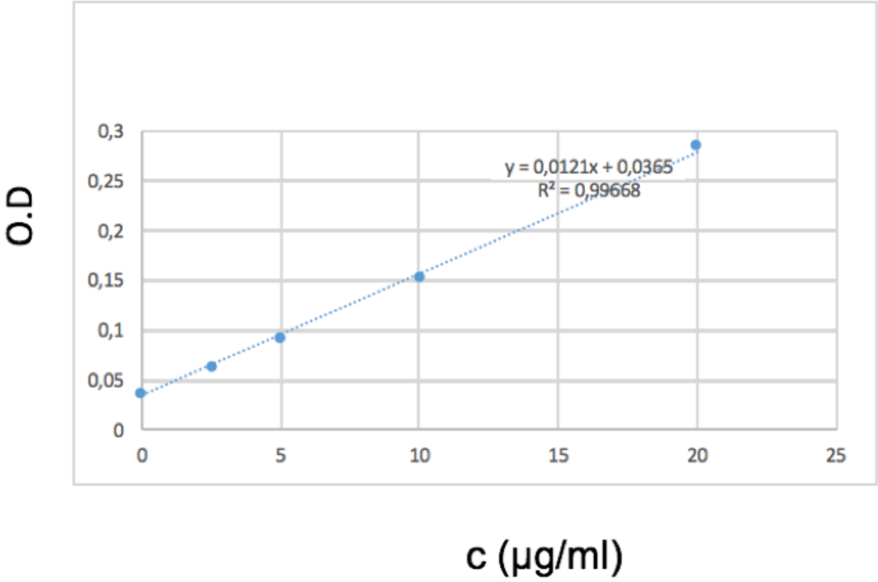

Figure S6: Standard calibration curve of absorption at 499 nm of Doxorubicin in PBS.

4. The concentration of unknown Doxorubicin or Tamoxifen sample can be easily determined once we have its absorption value at the characteristic

wavelength, using the standard calibration formula obtained by linear regression from the data in Table 1, 2 or 3. Having these results, we can further calculate the encapsulation efficiency or release percent by comparing the measured concentration to the initial drug feeding concentration.

5. Example of absorbance spectra of nanoparticles loaded with Doxorubicin.

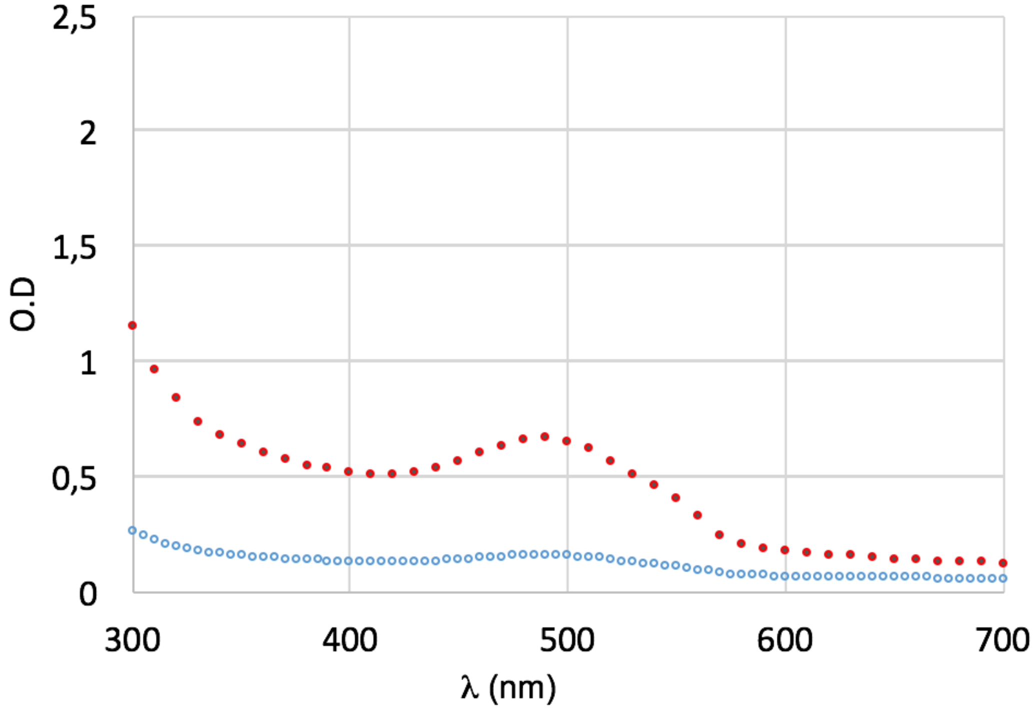

Figure S7: The filled circles correspond to a spectrum recorded in a cuvette of thickness 1 cm, the open one to a spectrum recorded in a cuvette of thickness 1mm. The spectra display a peak at 481 nm suggesting that the drug is in less polar environment than in water and thus well encapsulated in nanoparticles. We recall that the peak for Doxorubicin in water is at 499 nm whereas the one in DMSO is at 481 nm. These NPs have a hydrodynamic diameter of 138 nm (PDI=0.27) therefore the baseline ascribed to light scattering is moderate and, in principle, it can be subtracted in order to get the contribution of Doxorubicin absorption only. However, we preferred to perform a disruptive measurement (dissolving the NPs in DMSO to obtain a flat baseline and thus more precisely obtain the drug concentration from the regression law of Figure S2. We recall that the presence of PLGA molecules in concentration lower than 200 μg/ml do not change the absorbance of pure DMSO.

6. Measurement of interfacial tension between water and a DCM-DMSO mixture by the pendant drop weighting method.

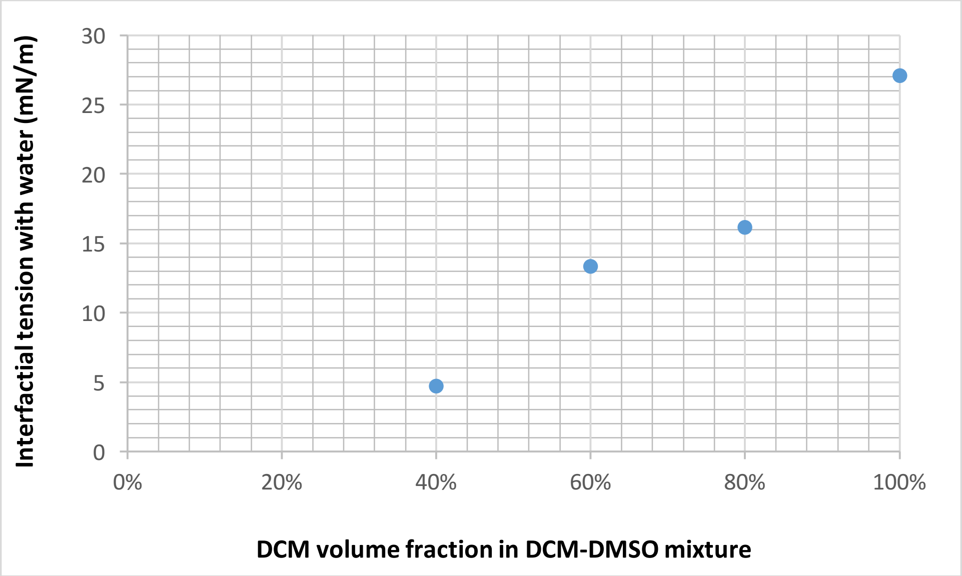

Figure S8: Interfacial tension measured between a DCM-DMSO mixture and water.
